# GUN4 Affects the Circadian Clock and Seedlings Adaptation to Changing Light Conditions

**DOI:** 10.3390/ijms23010194

**Published:** 2021-12-24

**Authors:** Tao Li, Rui Wu, Zhixin Liu, Jiajing Wang, Chenxi Guo, Yaping Zhou, George Bawa, Xuwu Sun

**Affiliations:** 1 State Key Laboratory of Crop Stress Adaptation and Improvement, School of Life Sciences, Henan University, 85 Minglun Street, Kaifeng 475001, China; litaossd@126.com (T.L.); wuruiwr347538@sina.com (R.W.); zxlsch2019@163.com (Z.L.); wdj_3079@sina.com (J.W.); chenxi1445@163.com (C.G.); zhouyapinghenu@sina.com (Y.Z.); ge.9410@yahoo.com (G.B.); 2 State Key Laboratory of Cotton Biology, School of Life Sciences, Henan University, 85 Minglun Street, Kaifeng 475001, China; 3 Key Laboratory of Plant Stress Biology, School of Life Sciences, Henan University, 85 Minglun Street, Kaifeng 475001, China

**Keywords:** *gun4*, medium-strong light, adaptation, circadian clock

## Abstract

The chloroplast is a key organelle for photosynthesis and perceiving environmental information. GENOME UNCOUPLED 4 (GUN4) has been shown to be required for the regulation of both chlorophyll synthesis, reactive oxygen species (ROS) homeostasis and plastid retrograde signaling. In this study, we found that growth of the *gun4* mutant was significantly improved under medium strong light (200 μmol photons m^−2^s^−1^) compared to normal light (100 μmol photons m^−2^s^−1^), in marked contrast to wild-type (WT). Further analysis revealed that GUN4 interacts with SIGNAL RECOGNITION PARTICLE 54 KDA SUBUNIT (SRP43) and SRP54. RNA-seq analysis indicated that the expression of genes for light signaling and the circadian clock is altered in *gun4* compared with (WT). qPCR analysis confirmed that the expression of the clock genes *CLOCK-RELATED 1* (*CCA1*), *LATE ELONGATION HYPOCOTYL* (*LHY*), *TIMING OF CAB EXPRESSION 1* (*TOC1*) and *PSEUDO RESPONSE REGULATOR 7* (*PRR7*) is significantly changed in the *gun4* and *srp54* mutants under normal and medium strong light conditions. These results suggest that GUN4 may coordinate the adaptation of plants to changing light conditions by regulating the biological clock, although it is not clear whether the effect is direct or indirect.

## 1. Introduction

Chloroplasts are essential organelles for both photosynthesis and metabolic reactions [1]. During the long evolution process, more than 95% of the genes encoding chloroplast proteins were integrated into the nucleus, and only a small number remained in the chloroplast genome. It contains about 100–250 genes in different species [2]. Therefore, to ensure the correct functioning of chloroplasts during development and under changing environmental conditions, it is necessary to coordinate the activity of the nucleus and chloroplast genome through intracellular signaling [3]. This communication between nucleus and chloroplast is bidirectional [3,4]. Anterograde signaling occurs from the nucleus to chloroplast to regulate the development of chloroplasts [4,5]; signaling from chloroplasts to the nucleus, called retrograde signaling, regulates the expression of nuclear genes coding for specific chloroplast factors [6,7,8]. Five genomes uncoupled (gun) mutants defective in plastid retrograde signaling have been previously identified in *Arabidopsis* [3,4,9], among which the *gun4* mutant was defective in growth and chlorophyll biosynthesis as revealed by the appearance of yellow leaves [10,11]. GUN4 only exists in organisms undergoing oxygenic photosynthesis [12]. In addition, plastid retrograde signals regulate many other biological processes in plants, such as the biological clock and medium-strong light adaption [13,14].

The biological clock network includes a transcription-translation feedback loop controlled by environmental signals, such as light and temperature, to adjust the rhythm phase to the local environment [15]. The positive and negative feedback loop includes the positive element TIMING OF CAB EXPRESSION 1 (TOC1) [16,17], which is responsible for activating the expression of two single MYB proteins, CLOCK-RELATED 1 (CCA1) [18] and LATE ELONGATION HYPOCOTYL (LHY) [19]. These MYB proteins act as negative elements in the cycle and inhibit the activity of positive elements [20]. The resulting activation and inhibition cycle may require several unknown modification steps [21]. Subsequently, other feedback loops identified indicate that the oscillator may be composed of multiple interlocking feedback loops to ensure that the circadian rhythm system operates accurately under various environmental conditions [22].

Soluble sugars produced by photosynthesis, especially sucrose and glucose, are the key metabolites of the plant biological clock [23,24]. It was found that the endogenous oscillation of sugar level provides metabolic feedback to the circadian oscillator through the morning-expressed gene PSEUDO RESPONSE REGULATOR 7 (PRR7). It was also found that the *prr7* mutant is not sensitive to the sucrose circadian rhythm cycle [13]. Therefore, photosynthesis plays a significant role in the entrainment and maintenance of the circadian rhythm in *Arabidopsis*.

In addition, the interaction between reactive oxygen species (ROS) production and photosynthetic metabolism is important in plants [25]. The control of endogenous ROS levels allows ROS to be used as signals and effectors to regulate growth, development, and stress responses [26]. The production of a series of key metabolites and reactive oxygen species in chloroplasts, including singlet oxygen (^1^O_2_), hydrogen peroxide (H_2_O_2_), superoxide anion (O_2_^−^) and hydroxyl radical (OH·), leads to changes in chloroplast redox state and perturbs cellular metabolic functions [26]. These effects are also related to redox signals, ROS, light, stress, and regulation of photosynthetic electron transport [27]. The protective effect of soluble sugar may occur during the normal circadian cycle of sugar accumulation and utilization in plants [28]. GUN4 is essential for protecting plants against photooxidation by binding free porphyrins under light which limits the accumulation of ROS *in vivo* [29]. Therefore, GUN4 possibly acts as a key regulator in maintaining sugar production during photosynthesis and coordinating sugar and ROS balance.

To further explore the role of GUN4 in regulating plant growth and development, we conducted a systematic characterization of the *gun4* mutant. Our results indicate that GUN4 interacts directly with SRP43 and SRP54. SRP43 and SRP54 can form a transient complex to regulate the light-harvesting antenna complex (LHC) assembly. The SRP43, as an ATP-independent chaperone, interacts with SRP54 and is essential for protecting LHCPs from aggregation [30]. Recently the new role of SRP43 in the regulation of the tetrapyrrole biosynthesis has been characterized. SRP43 can positively regulate the stability of GluTR to enhance the synthesis of 5-aminolevulinic acid (ALA), which is subsequently used for chlorophyll (Chl) and heme production in *Arabidopsis*, respectively [31]. More recently, SRP43 was found to be involved in regulating the stability of CHLH and GUN4 during leaf greening and heat-shock stress [32]. In the *chaos* mutant, the SRP43 is inactivated and highly tolerant to photooxidative stress [33]. Changes in redox homeostasis may regulate the biological clock through the signaling pathway mediated by GUN4 to maintain chloroplast development and metabolic function under medium-strong light conditions. Taken together, our results suggest that GUN4 plays an important role in regulating, directly or indirectly, chloroplast redox homeostasis, chloroplast development and circadian rhythm.

## 2. Results

### 2.1. GUN4 Interacts with SRP43 and SRP54

**Figure 1:**
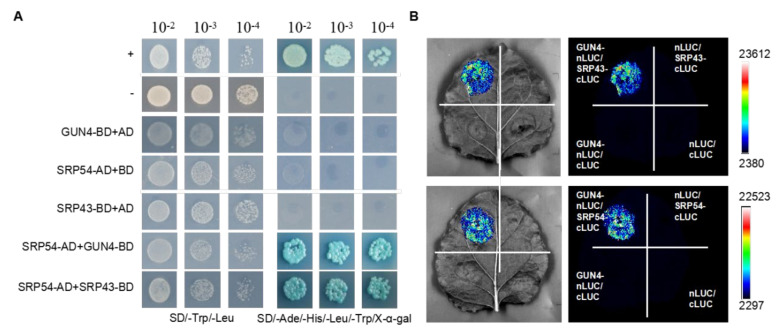
Analysis of the interaction between GUN4 and interactors by yeast two-hybrid and BiLC. (**A**) Growth of clones co-expressing the indicated vector combinations on SD/-Trp/-Leu and SD/-Trp/-Leu/-Ade/-His/X-α-Gal. AD: Gal4 DNA-binding domain; BD: Gal4 transciptional activation domain; SD: synthetically defined medium. (**B**) Agrobacteria containing the indicated vector combinations were co-inoculated into tobacco leaves. The fluorescence signals of luciferase were detected by a CCD image camera (tanon 5200). LUC: Luciferase. Signal intensities correspond to the color scale at the right of the panel.

The chloroplast signal recognition particle (cpSRP) composed of the SRP43 and SRP54 subunits is involved in LHCB protein assembly and plays a vital role in chloroplast stromal sorting and transport to the thylakoid membrane [34,35,36]. LHCB proteins bind chlorophylls. The phenotype of *chaos* (*srp43*) and *srp54* mutants was similar to that of the *gun4* mutant with regard to defects in chloroplast development [32,36]. Therefore, we speculated that there may be a functional interaction between SRP43, SRP54 and GUN4. To determine whether there was a functional relationship between GUN4, SRP43 and SRP54, we first studied their interaction by yeast two-hybrid assays (Y2H). The results revealed that SRP54 interacts with GUN4 and SRP43 (Figure 1A). In addition, bimolecular luciferase complementation (BiLC) was performed to verify the interaction *in vivo* detected by Y2H. Strong fluorescence interaction signals were observed when GUN4-nLUC and SRP43-cLUC and GUN4-nLUC and SRP54-cLUC were co-transformed. As negative controls, no fluorescence signals were detected with the combinations of GUN4-nLUC and cLUC, nLUC and SRP43 cLUC, nLUC and SRP54 cLUC, and nLUC and cLUC (Figure 1B). The interaction between GUN4 and SRP43 has also been confirmed by co-immunoprecipitation assays [32].

### 2.2. GUN4 Is Involved in Regulating ROS Homeostasis and Seedling Development

**Figure 2:**
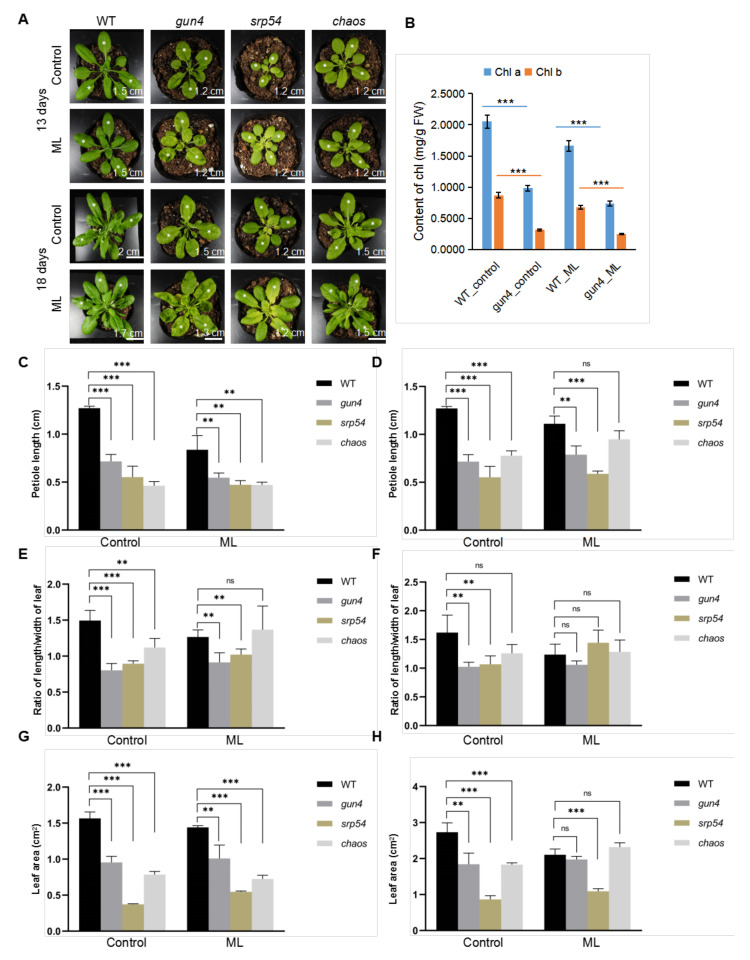
**(A)** Phenotype of WT and mutants grown in the soil. Seven-day-old seedlings of wild-type (WT), *gun4*, *srp54* and *chaos* (*srp43*) were transferred to soil for further growth for 13 and 18 days, respectively. The leaves labeled with white stars were used for statistical analysis of the leaf area, petiole length and ratio of length/width of leaf. Control: normal light (100 μmol photons m^−2^s^−1^); ML: medium strong light (200 μmol photons m^−2^s^−1^). (**B**) Analysis of the content of chlorophyll a and b. (**C**) and (**D**) Statistical analysis of the petiole length of seedlings of WT and mutants. (**E**) and (**F**) Statistical analysis of the leaf area of seedlings of WT and mutants. (**G**) and (**H**) Statistical analysis of the ratio of length/width of the leaf of WT and mutant seedlings. The data were analyzed by one-way ANOVA following Brown-Forsythe test. ns: *p* > 0.05, **: *p* < 0.01, ***: *p* < 0.001.

GUN4 interacts with cpSRP54 and cpSRP43, which suggests a functional link between them. Therefore, we first analyzed the growth phenotype of mutants affected by these genes in soil. The yellow rosette leaves of the *gun4* mutant are due to deficiencies in chlorophyll biosynthesis and chloroplast development (Figure 2). When grown in soil, compared with wild-type (WT), the *gun4* mutant grew slower, had shorter stem length, shorter petiole and a lower length-width ratio of the leaf (Figure 2). Compared with normal light conditions (100 μmol photons m^−2^s^−1^, 12 h light/12 h dark), the growth rate of *gun4* and *srp54* mutants increased under medium-strong light (200 μmol photons m^−2^s^−1^, 12 h light/12 h dark) (Figure 2), suggesting that medium-strong light may improve their growth and development to some extent. In conclusion, GUN4 not only regulates chlorophyll biosynthesis but also affects overall plant growth and development.

### 2.3. Identification of the Target Genes Regulated by GUN4 Using RNA-seq

**Figure 3:**
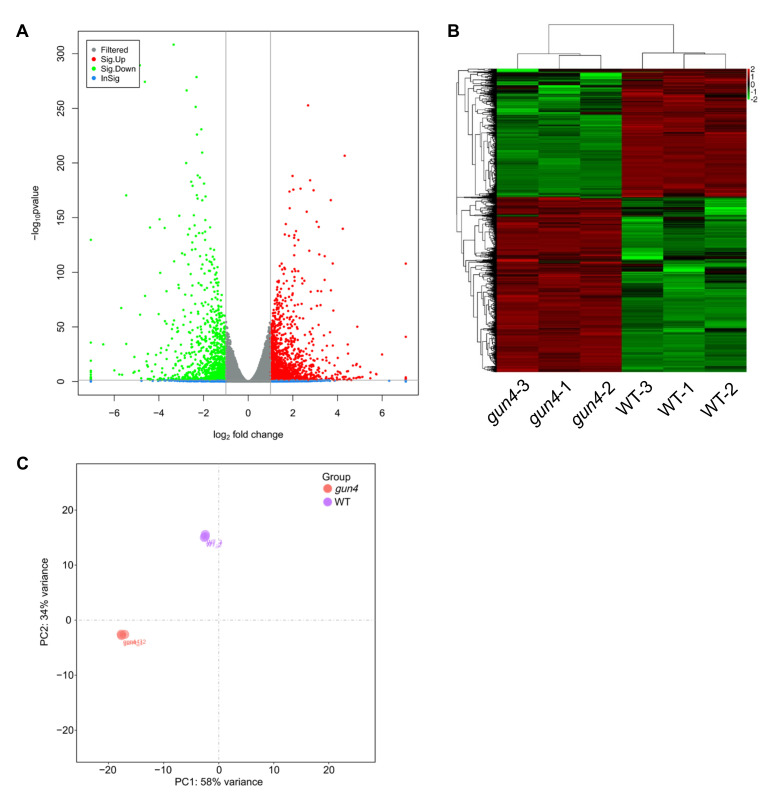
RNA-seq analysis of gene expression profiles in seedlings of WT and *gun4* mutant under normal growth conditions. (**A**)Volcano plot shows the expression of differentially expressed genes in *gun4*. (**B**) Heatmap of differentially expressed genes in *gun4*. (**C**) Principal component analysis (PCA) of gene expression in each sample.

**Figure 4:**
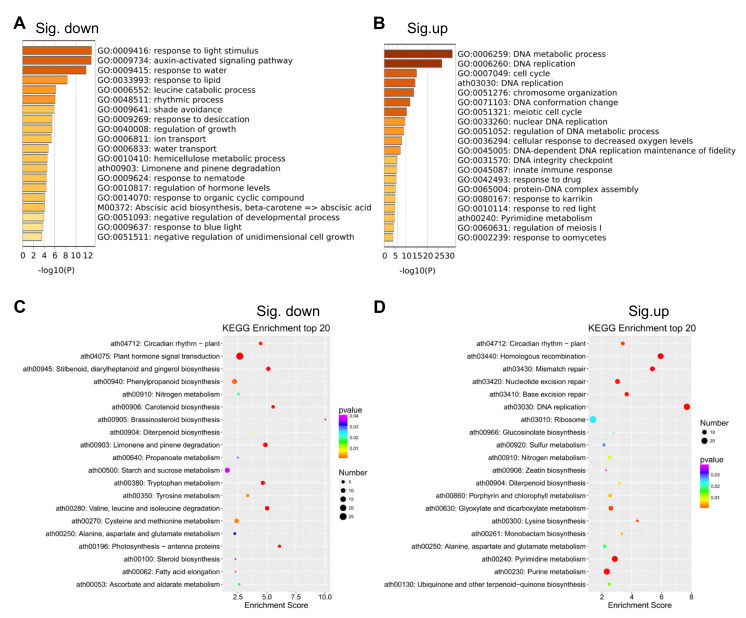
Gene ontology (GO) and Kyoto Encyclopedia of Genes and Genomes (KEGG) analysis of differentially expressed genes in *gun4.* (**A**,**B**) Top 20 GO terms of differentially expressed genes in *gun4*. (**C**,**D**) Top 20 KEGG terms of differentially expressed genes in *gun4*.

GUN4 is involved in regulating the expression of nuclear genes and Mg^2+^ chelatase activity in the chlorophyll biosynthesis pathway [37,38,39]. Important progress on the mechanisms underlying the regulation of chlorophyll synthesis by GUN4 has been achieved [10,40,41]. However, little is known about how GUN4 affects the expression of nuclear genes. To this end, we performed RNA-seq to compare the transcriptomes of *gun4* and wild-type. This comparison revealed 1282 differentially expressed genes (DEGs) in *gun4*, of which 734 and 548 genes were significantly up-and down-regulated, respectively (Figure 3 and Supplementary Materials Table S1). PCA analysis of gene expression levels in each sample showed no significant difference in gene expression among the three biological replicates (Figure 3). Gene ontology (GO) enrichment analysis showed that in the *gun4* mutant, the down-regulated genes are mainly related to light signaling, auxin activation signaling, water transport, rhythmic processes and shade avoidance (Figure 4A). In contrast, the up-regulated genes in *gun4* are predominantly involved in DNA metabolism, DNA replication, chromosome organization, meiosis, cell cycle and the innate immune response (Figure 4B). The Kyoto Encyclopedia of Genes and Genomes (KEGG) analysis showed that the down-regulated genes in *gun4* are mainly implicated in regulating the plant circadian rhythm, hormone signal transduction, carotenoid biosynthesis and starch and sucrose metabolism (Figure 4C), whereas the up-regulated genes are principally involved in plant circadian rhythm and porphyrin and chlorophyll metabolism. (Figure 4D). For example, compared with WT, in *gun4*, expression of the circadian clock-related genes *CCA1*, *LHY*, *CYCLING DOF FACTOR 1* (*CDF1*), *PSEUDO-RESPONSE REGULATOR 7* (*PRR7*), *PRR9* and *CONSTANS-LIKE 1* (*COL1*) are down-regulated, while expression of *PRR1*, *PRR3*, *EARLY FLOWERING 3* (*ELF3*), *ELF4* and *CONSTANS* (*CO*) are up-regulated (Figure S1A). These results point to a possible role of GUN4 in the regulation of the plant circadian rhythm. Interestingly, we also found that the genes involved in light signaling are significantly down-regulated in *gun4* (Figure S1B). These observations suggest that GUN4 may participate in the synergy between light signaling and circadian rhythm to regulate nuclear gene expression and possibly help plants to synchronize their growth and development with the circadian clock.

### 2.4. Analysis of the Biological Clock under Medium-Strong Light Conditions

**Figure 5:**
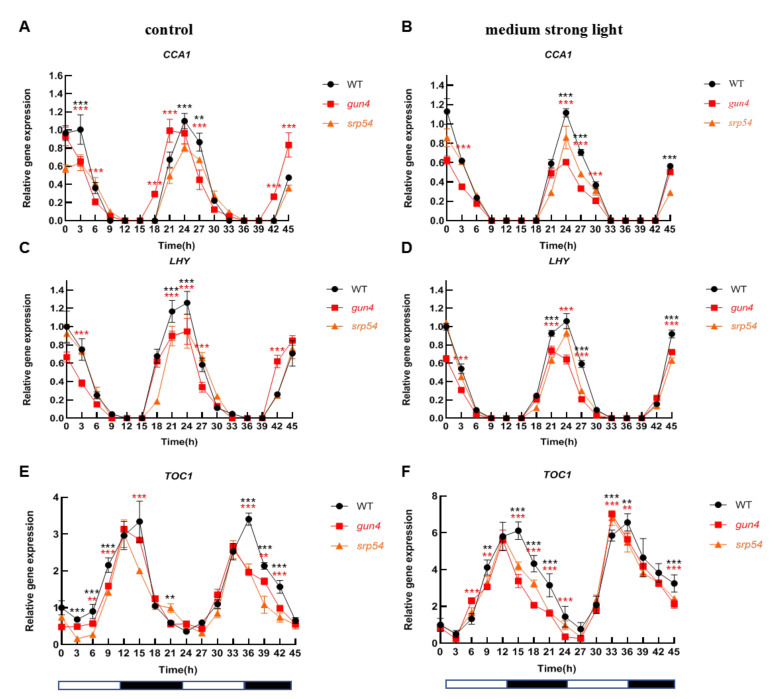
Analysis of the rhythmic patterns of *CCA1*, *LHY*, and *TOC1*. Under normal light and medium-strong light conditions, leaf samples of 2-week-old WT, *gun4*, and *srp54* mutant seedlings were taken at different times every 3 h as indicated. A total of 16 samples were taken. Total RNA was extracted, reverse transcribed into cDNA and the expression of annotated genes was estimated by qPCR. *Actin* was used as an internal control for normalization. (**A**), (**C**), and (**E**) Analysis of the relative expression of *CCA1*, *LHY*, and *TOC1* under normal light conditions, respectively. (**B**), (**D**), and (**F**) Analysis of the relative expression of *CCA1*, *LHY*, and *TOC1* under medium strong light conditions, respectively. Relative expression indicates the mean value (±SD) of three independent experiments. White bars: light; black bars: dark. The red stars represent student’s *t*-test of *gun4* versus WT. The black stars represent student’s *t*-test of *srp54* versus WT. **: *p* < 0.01, ***: *p* < 0.001.

**Figure 6:**
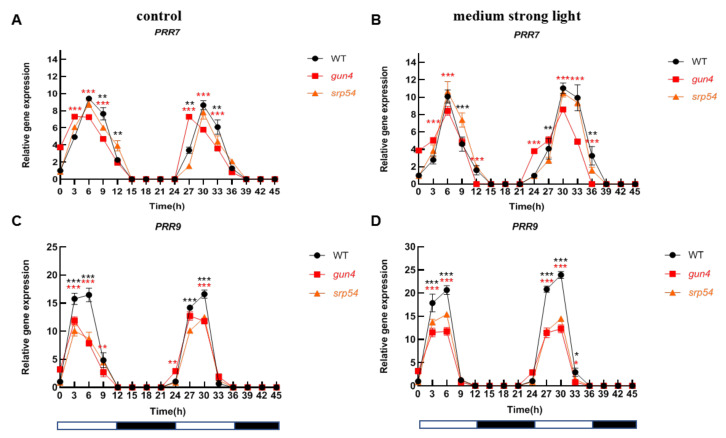
Analysis of the rhythmic patterns of *PRR7* and *PRR9*. Under normal light and medium-strong light conditions, leaf samples of 2-week-old WT, *gun4*, and *srp54* mutant seedlings were taken at different times every 3 h as indicated. A total of 16 samples were taken. Total RNA was extracted, reverse transcribed into cDNA, and the expression of annotated genes was estimated by qPCR. *Actin* was used as an internal control for normalization. (**A**) and (**C**) Analysis of the relative expression of *PRR7* and *PRR9* under normal light conditions, respectively. (**B**) and (**D**) Analysis of the relative expression of *PRR7* and *PRR9* under medium strong light conditions, respectively. Relative expression indicates the mean value (±SD) of three independent experiments. White bars: light; black bars: dark. The red stars represent student’s *t*-test of *gun4* versus WT. The black stars represent student’s *t*-test of *srp54* versus WT. *: *p* < 0.05, **: *p* < 0.01, ***: *p* < 0.001.

Considering the important role of GUN4 in regulating ROS homeostasis in chloroplasts and that medium-strong light treatment causes an increase of ROS in chloroplasts, we speculated that GUN4 may be involved in the optimal maintenance of the circadian rhythm under medium-strong light conditions. Considering the interaction between GUN4 and SRP54 and their similar response to medium-strong light, we analyzed the biological rhythm of expression of several clock genes in the *gun4* and *srp54* mutants under normal light and medium-strong light. As shown in Figure 5A,C, under normal growth conditions (12 h light/12 h dark), the level of *CCA1* and *LHY* in WT decreased from the 0-time point (onset of light period) until time point 12. Subsequently, the level increased rapidly until time point 21 before entering the next cycle. A similar circadian pattern for CCA1 in WT, *gun4* and *srp54* mutants was detected under continuous dark conditions (Figure S2), suggesting that the effects of *gun4* on the expression of *CCA1* existed under continuous dark conditions. Accumulation of *TOC1* showed an opposite pattern to that of *CCA1* and *LHY* (Figure 5E). Medium-strong light had a significant effect on the expression of the core components of the biological clock. Under normal light conditions, the expression of *PRR7* and *PRR9* showed a similar rhythmic pattern. As shown in Figure 6A, the expression of *PRR7* increased rapidly after the light was turned on until it reached the maximum at time point 6. Then, its expression decreased rapidly and reached its lowest value at time point 15. At time point 21, its level started to rise again and followed the rhythmic cycle. Interestingly, we found that under normal light conditions, compared with WT, *PRR7* reached its peak 3 h earlier at time point 27 in *gun4* (Figure 6A). Under the same conditions, compared with WT, the peak of *PRR9* expression in *srp54* and *gun4* was 3 h earlier (Figure 6C). Under medium-strong light, the expression of *PRR7* and *PRR9* was the same as in WT and peaked at time point 6 (Figure 6B,D). These results show that the level of irradiance strongly affects the biological rhythm of *PRR7* and *PRR9* expression in *gun4*.

In addition, we also analyzed the expression pattern of *ELF3*, *ELF4*, and *CO* in detail. Overall, *ELF3* and *CO* showed similar rhythmic patterns, while *ELF4* expression displayed a relatively unique pattern (Figure S3). Under medium-strong light conditions, *CO* maintained rhythmic fluctuations similar to those under normal light conditions, while the rhythm amplitude of *ELF3* increased, the rhythm amplitude of *ELF4* decreased (Figure S3). These results suggest that *ELF3* and *ELF4* expressions are most affected by the medium-strong light treatment.

It is well known that the expression of a large number of genes of chloroplast proteins shows obvious rhythmic fluctuations. Therefore, we also measured the rhythmic pattern of representatives of these genes, as shown in Figure S3. Under normal light conditions, *GOLDEN2 LIKE 2* (*GLK2*) and *LIGHT HARVESTING CHLOROPHYLL A/B BINDING PROTEIN* *2.1* (*LHCB2.1*) showed obvious rhythmic fluctuations. Overall, the rhythmic fluctuations of *GLK2* occurred earlier than *LHCB2.1* (Figure S4A,C,E). Under medium-strong light conditions, the expression of *GLK2* was higher (Figure S4D,F). Although medium-strong light treatment affects the expression of *GLK2* and *LHCB2.1*, it does not affect their rhythmic expression. Compared to WT, the peak of expression in *gun4* and *srp54* of *GLK2* and *LHCB2.1* occurred 6 and 3 h earlier, respectively (Figure S4C,E). Comparative analysis of *LHCB2.1* expression under normal and medium-strong light conditions showed that medium-strong light mainly changed the expression pattern of *LHCB2.1* rather than the expression level (Figure S5). To determine whether the expression of *GUN4* itself is rhythmic, we also analyzed its expression pattern and that of *GUN5*. As shown in Figure S6, both *GUN4* and *GUN5* showed some rhythmic patterns under normal light conditions, although the amplitude of the fluctuations of *GUN5* was relatively small. In general, *GUN4*, *GLK2* and *LHCB2.1* all showed similar rhythmic fluctuations (Figure S4 and Figure S6), pointing to some synchronicity between them.

Interestingly, compared with normal light conditions, medium-strong light treatment enhanced the rhythm amplitude of *GUN4* (Figure S6A,B) but decreased the expression of *GUN5* (Figure S6C,D). In addition, we also analyzed the biological rhythm of *SRP54*. Under medium-strong light conditions, the expression of *SRP54* displayed fluctuations without any obvious rhythm (Figure S7).

### 2.5. GUN4 Affects the Expression of Genes of the Biological Clock

Because GUN4 affects the expression of core components of the biological clock, we further analyzed the effect of GUN4 on the rhythm of nuclear gene expression. As shown in Figure 5A,C,E, under normal light conditions, compared with WT, the rhythmic phase of *CCA1* and *TOC1* expression in *gun4* was significantly earlier, while the rhythmic phase of *LHY* expression was almost the same in WT. Under medium-strong light conditions, expression of *CCA1* and *LHY* in *gun4* mutant was slightly decreased, suggesting that GUN4 may impact the rhythmic expression of the core components under medium-strong light conditions.

The analysis of biological rhythms of *PRR7* and *PRR9* showed that under normal light conditions, the peak expression of *PRR7* and PRR9 in the *gun4* mutant occurred significantly earlier than in WT (Figure 6A,C). Under medium-strong light treatment, the peak expression of these two genes in WT and *gun4* was the same (Figure 6B,D). These results suggest that the effects of irradiance on the biological clock are impacted by GUN4.

Further analysis of chloroplast protein genes showed that under medium-strong light conditions, compared with WT, the expression level of *GLK2* in *gun4* decreased, while the expression of *LHCB2.1* increased slightly (Figure S4), pointing to a possible role of GUN4 in regulating the rhythmic expression of chloroplast protein genes.

## 3. Discussion

### 3.1. GUN4 Is Required for Optimal Growth and Development of Seedlings

The GUN4 protein is a porphyrin binding protein encoded by the nucleus and located in the chloroplast [10,29]. It participates in chlorophyll biosynthesis by activating Mg-chelatase [10]. SRP54 and SRP43 are involved in the assembly of LHCB in the thylakoid membrane [34,35,36]. LHCB is a protein binding chlorophyll that captures light energy for photosynthesis. GUN4 is also required for preventing ROS production caused by free porphyrins under light [29]. Considering the close functional relationship between GUN4, SRP43 and SRP54, we first analyzed whether they directly interact with each other. Our results indicate that SRP54 interacts with GUN4 and SRP43 (Figure 1). In addition, recent studies also revealed a physical interaction between GUN4 and SRP43 [32], which agrees with our results (Figure 1).

Under normal light conditions, compared with WT, the leaves of *gun4* mutant seedlings are yellowish (Figure 2). GO enrichment analysis of our transcriptomic data indicated that the expression of genes for carotenoid biosynthesis, plant circadian rhythm, starch and sucrose metabolism and porphyrin and chlorophyll metabolism changed significantly in the *gun4* mutant compared with WT (Figure 4). These results suggest that GUN4 may be involved in plant growth and development and circadian rhythm.

### 3.2. Is GUN4 Involved in Regulating the Biological Clock?

**Figure 7:**
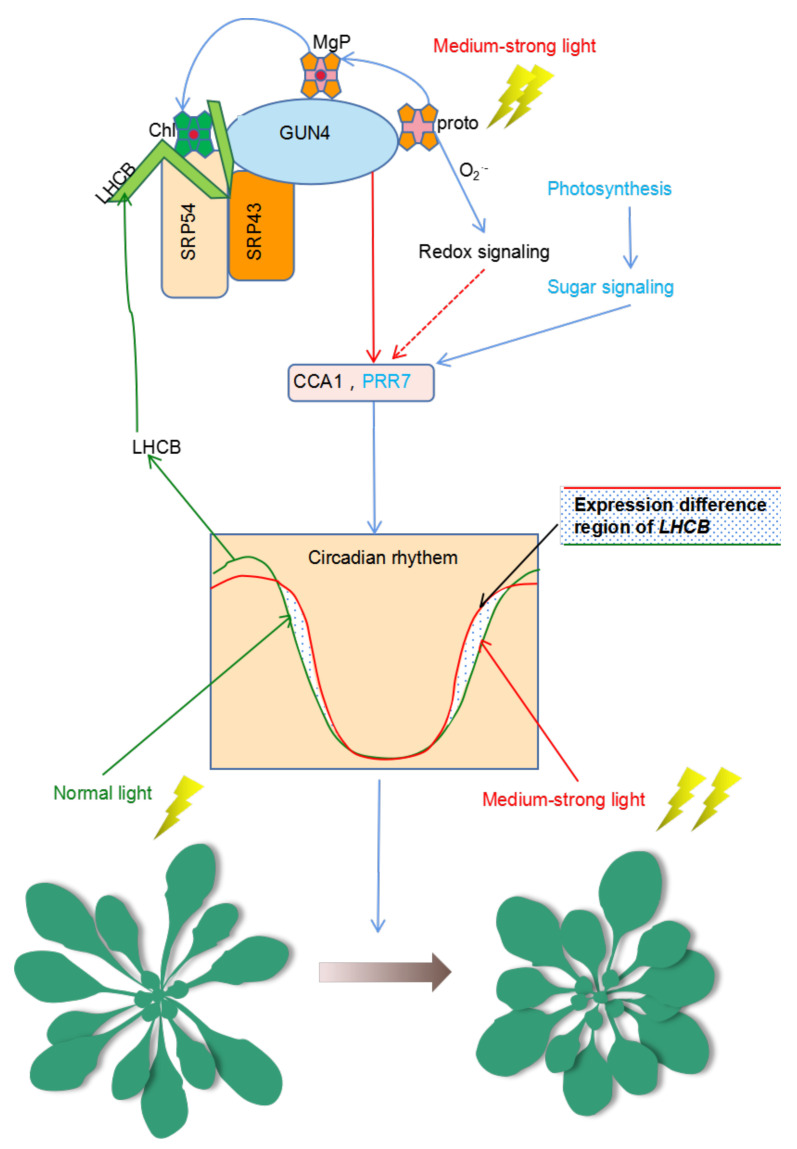
Model for the dual participation of GUN4 in the regulation of homeostasis of ROS and the biological clock. Under normal light conditions, GUN4 can interact with SRP43 and SRP54 to coordinate the assembly of LHCB protein and the binding of newly synthesized chlorophyll molecules to promote photosynthesis and sugar production. GUN4 affects the expression of CCA1 and PRR7, the core components of the biological clock as well as the expression of nuclear genes of chloroplast proteins such as LHCB. Under medium-strong light, the balance between ROS production and scavenging is compromised, resulting in ROS accumulation and the activation of redox signals that modulate the circadian expression of *LHCB*. Changes in the expression of *LHCB* and other genes may induce changes in seedling development. Possibly, GUN4 may regulate the adaptation of plants to medium-strong light through its action on the biological clock together with redox signals. Protoporphyrin IX (Proto); Mg-Proto (MgP); Chlorophyll (Chl).

In *Arabidopsis*, the circadian clock regulatory network comprises multiple complex positive and negative feedback regulatory loops, which maintain a circadian cycle of nearly 24 h (Figure 7). Analysis of the expression of *CCA1*, *LHY* and *TOC1* indicated that their rhythmic mRNA levels in *gun4* were significantly changed compared with WT (Figure 5). Under normal light conditions, the peak of *PRR7* and PRR9 transcripts in *gun4* occurred 3 to 6 h earlier than in WT but not under medium strong light conditions (Figure 6A,C). Previous studies have shown that PRR7 regulates the biological rhythm of chloroplast sugar signals [13]. We found that the expression level of *PRR7* and *PRR9* in *gun4* was significantly decreased compared with WT (Figure 6A,B). In addition, under medium-strong light conditions, compared with WT, the expression of *ELF3* and *CO* in *gun4* increased (Figure S3B,D,F) while *ELF4* expression decreased, and the corresponding rhythmic patterns slightly changed, suggesting some influence of GUN4. Overall, compared with WT, the rhythm patterns of some marker genes (e.g., TOC1 and PRR9) showed similar changes between GUN4 and SRP54 mutants, while others (e.g., CCA1, LHY and PRR7) displayed inconsistent trends between gun4 and srp54 mutants. One possible explanation is that GUN4, as a key regulator of plastid retrograde signaling, plays a major role in regulating the expression of these genes, and SRP54 may indirectly affect the regulation of GUN4 on the expression of these genes through its interaction with GUN4. In addition to the genes involved in regulating the clock, we also determined the rhythmic expression of genes related to chloroplast development in *gun4* and *srp54* mutants. Under normal light conditions, *GLK2* and *LHCB2.1* showed similar rhythm patterns in WT, *gun4*, and *srp54* although the peak of expression of *GLK2* was reached slightly earlier than WT (Figure S5). However, the expression patterns of *GLK2* and *LHCB2.1* changed in WT under medium-strong light conditions, and significant differences were observed for *gun4* and *srp54* (Figure S5B,D and Figure 7), indicating that the loss of GUN4 and SRP54 affects the expression of *GLK2* and *LHCB2.1* under medium-strong light conditions, although it is not clear whether these effects are direct or indirect as a result of changes in chlorophyll metabolism.

## 4. Materials and Methods

### 4.1. Plant Materials and Growth Conditions

Wild-type (WT) *Arabidopsis* (*Arabidopsis thaliana*) Columbia ecotype (Col-0) was used in this study. Mutants in the Col-0 background were obtained from the Arabidopsis Biological Resource Center (ABRC) (Supplementary Materials Table S2). All mutants and WT *Arabidopsis* and *Nicotiana benthamiana* were grown in an artificial climate chamber under the growth conditions of 21–23 °C, 100 μmol photons m^−2^s^−1^ (normal light treatment), 12 h light/12 h dark, 60–70% humidity. For medium-strong light treatment, the seedlings were grown in an artificial climate chamber under the growth conditions of 21–23 °C, 200 μmol photons m^−2^s^−1^, 12 h light/12 h dark, 60–70% humidity.

### 4.2. Construction and Detection of Yeast Two-Hybrid Vectors

Full-length cDNA fragments of *GUN4*, *SRP54* and *SRP43* were amplified by PCR with corresponding primers (Supplementary Materials Table S3). The cDNA fragments were inserted into pBGKT7 and pGADT7 to produce GUN4-BD, SRP54AD, SRP54-BD and SRP43-BD, respectively. Subsequently, plasmids containing corresponding interacting partners were co-transformed into the yeast Y2H Gold strain. The interaction was considered to be positive when the colonies turned blue on SD/-Trp/-Leu/-Ade/-His/x-α-Gal plates.

### 4.3. Bimolecular Luciferase Complementation Experiment

Full-length cDNA fragments of *GUN4*, *SRP54* and *SRP43* were amplified by PCR with corresponding primers (Supplementary Materials Table S4). The resulting cDNA fragments were inserted into pCAMBIA 1300-nLUC and pCAMBIA 1300-cLUC to produce GUN4-nLUC, SRP43-cLUC and SRP54-cLUC, respectively. Then, these plasmids were transformed into Agrobacterium strain GV3101. The corresponding plasmids were co-injected into tobacco leaves for transient expression analysis. Three days later, after injection, the fluorescence signals of luciferase were detected by a CCD imager (tanon 5200).

### 4.4. RNA Extraction and qRT PCR

Total RNA was extracted with FastPure Plant Total RNA Extraction kit (Cat. No. DC104, Vazyme; Nanjing, China). Total RNA was treated with DNaseI (Vazyme; Nanjing, China) for 30 min to remove the remaining DNA, then the cDNA was synthesized with HiScript II One-Step RT-PCR Kit (Cat. No. P611, Vazyme; Nanjing, China); qRT-PCR was performed with the corresponding primers (Supplementary Materials Table S5). qPCR run was performed on a CFX 96 (Bio-Rad, Herculesm, CA, USA) with the following cycle parameter: 95 °C for 30 s, 35 cycles of 95 °C for 30 s, 55–56 °C for 15 s and 72 °C for 15 s.

### 4.5. RNA-seq

For RNA-seq, the leaves of 2-week-old seedlings of gun4 and WT were harvested at 10 o’clock in the morning and used for extracting the total RNA. Total RNA was extracted using the mirVana miRNA Isolation Kit (Ambion, Austin, TX, USA) following the manufacturer’s protocol. RNA integrity was evaluated using the Agilent 2100 Bioanalyzer (Agilent Technologies, Santa Clara, CA, USA). The samples with RNA Integrity Number (RIN) ≥ 7 were used for the subsequent analysis. The libraries were constructed using TruSeq Stranded mRNA LTSample Prep Kit (Illumina, San Diego, CA, USA) according to the manufacturer’s instructions. Then, these libraries were sequenced on the Illumina sequencing platform (HiSeqTM 2500 or Illumina HiSeq X Ten), and 125 bp/150 bp paired-end reads were generated. Raw data (raw reads) were processed using Trimmomatic [42]. The reads containing ploy-N and the low-quality reads were removed to obtain the clean reads. Then the clean reads were mapped to the reference genome using hisat2 [43]. Fragments Per Kilobase of exon model per Million mapped fragments (FPKM) [44] value of each gene was calculated using cufflinks [45], and the read counts of each gene were obtained by htseq-count [46]. DEGs were identified using the DESeq package (Available online: https://bioconductor.org/packages/release/bioc/html/DESeq2.html (accessed on 14 February 2020)). *p*-value < 0.05 and foldChange > 2 or foldChange < 0.5 was set as the threshold for significant differential expression.

### 4.6. Gene Ontology (GO) Enrichment Analysis

The enrichment of gene ontology (GO) terms and pathways for the DEGs were analyzed using Metascape (Available online: http://metascape.org/ (accessed on 10 October 2021)) [47].

## Data Availability

All data supporting the findings of this study are available within the paper and within its supplementary data published online.

## Supplementary Materials

The following supporting information can be downloaded at: https://www.mdpi.com/article/10.3390/ijms23010194/s1.
