# Supplementary material for: GUN4 Affects the Circadian Clock and Seedlings Adaptation to Changing Light Conditions
Source: Int J Mol Sci. 2021 Dec 24;23(1):194. doi: 10.3390/ijms23010194 (PMC8745339; doi:10.3390/ijms23010194)
Supplement: Supplementary file 1 [file ijms-23-00194-s001.zip › ijms-1462050-supplementary/Supplementary-sunxuwu-IJMS-20211204.pdf]

Supplementary Data

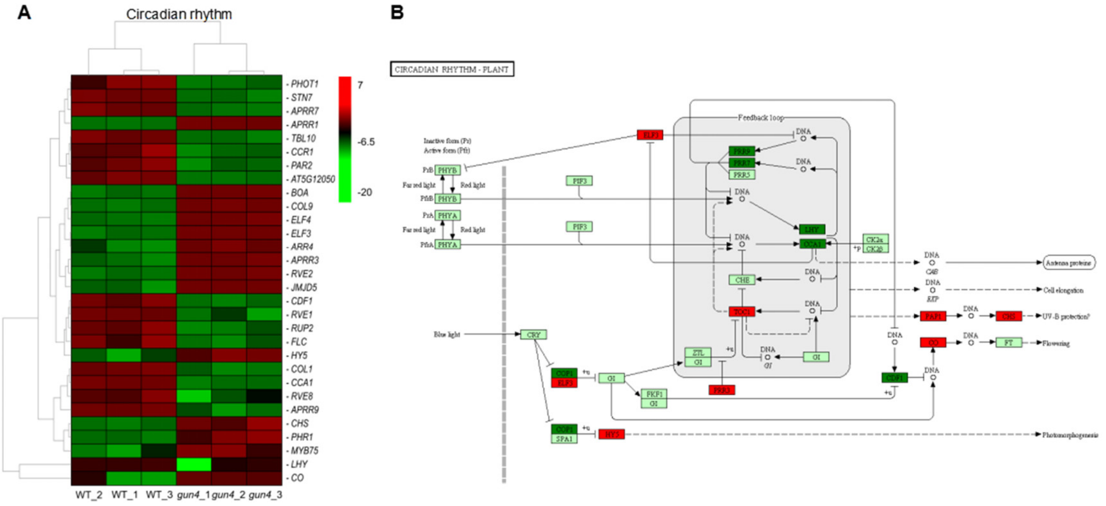

Supplementary Figure S1. Analysis of expression profiles of differentially expressed genes for circadian and red light signaling in *gun4* mutant.

(**A**) Heatmap analysis of circadian gene expression. (**B**) The expression patterns of clock marker genes in *gun4* in the model of KEGG. Red and green boxes represent up-and down-regulation of annotated genes, respectively.

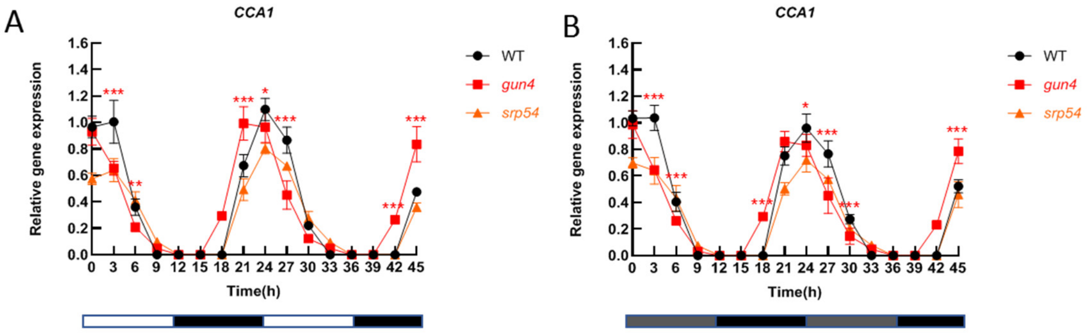

Supplementary Figure S2. Analysis of the rhythm patterns of *CCA1*.

Under normal light and continuous darkness conditions, leaf samples of 2-week-old WT, *gun4*, and *srp54* mutant seedlings were taken at different times every 3 hours as indicated. A total of 16 samples were taken. Total RNA was extracted, reverse transcribed into cDNA, and the expression of annotated genes was estimated by qPCR. *Actin* was used as internal control for normalization. Relative expression is given as the mean value (±SD) of three independent experiments. White bars: light; black bars: dark; gray bar: dark in the day. The red stars represent that student's t-test of *gun4* versus WT. *: p<0.05, **: p<0.01, ***: p<0.001.

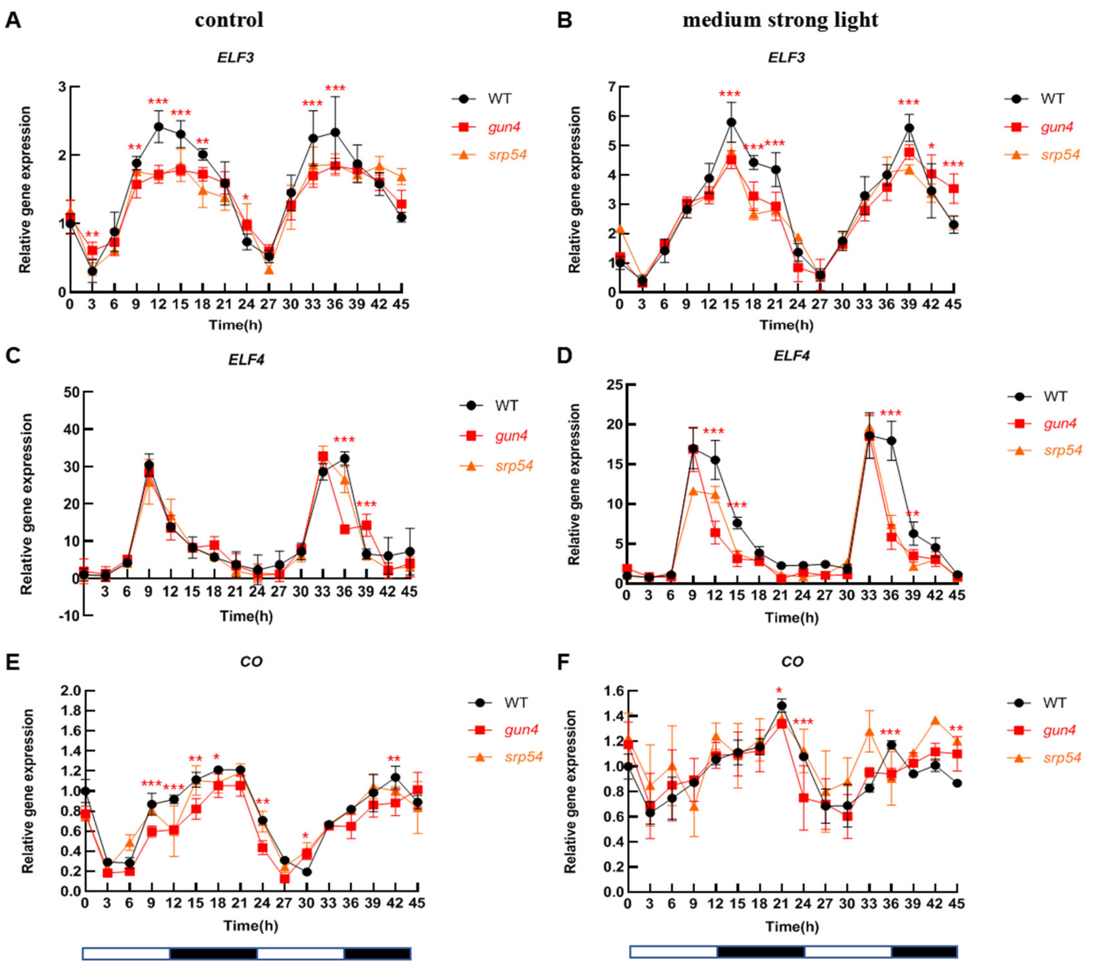

Supplementary Figure S3. Analysis of the rhythm patterns of *ELF3*, *ELF4,* and *CO.*

Under normal light and medium-strong light conditions, leaf samples of 2-week-old WT, *gun4*, and *srp54* mutant seedlings were taken at different times every 3 hours as indicated. A total of 16 samples were taken. Total RNA was extracted, reverse transcribed into cDNA, and the expression of annotated genes was estimated by qPCR. *Actin* was used as internal control for normalization. Relative expression is given as the mean value (±SD) of three independent experiments. White bars: light; black bars: dark. The red stars represent that student's t-test of *gun4* versus WT. *: $p<0.05$, **: $p<0.01$, ***: $p<0.001$.

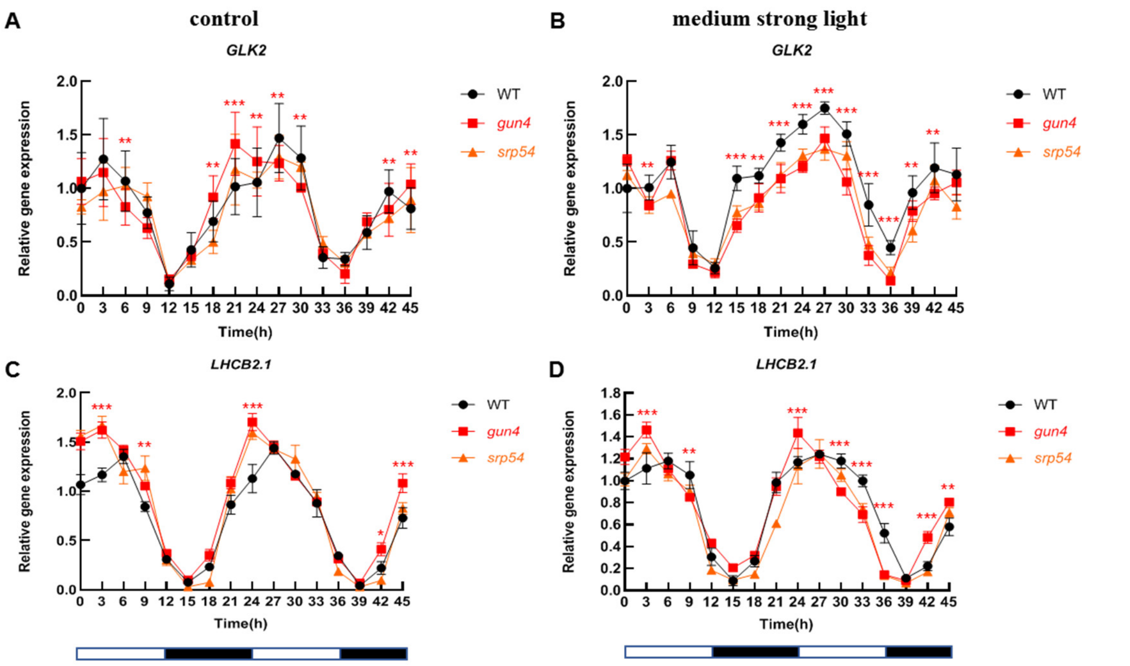

Supplementary Figure S4. Analysis of the rhythmic patterns of *GLK2* and *LHCB2.1.*

Under normal light and medium-strong light conditions, leaf samples of 2-week-old WT, *gun4*, and *srp54* mutant seedlings were taken at different times every 3 hours as indicated. A total of 16 samples were taken. Total RNA was extracted, reverse transcribed into cDNA, and the expression of annotated genes was estimated by qPCR. *Actin* was used as internal control for normalization. Relative expression is given as the mean value (±SD) of three independent experiments. White bars: light; black bars: dark. The red stars represent that student's t-test of *gun4* versus WT. *: p<0.05, **: p<0.01, ***: p<0.001.

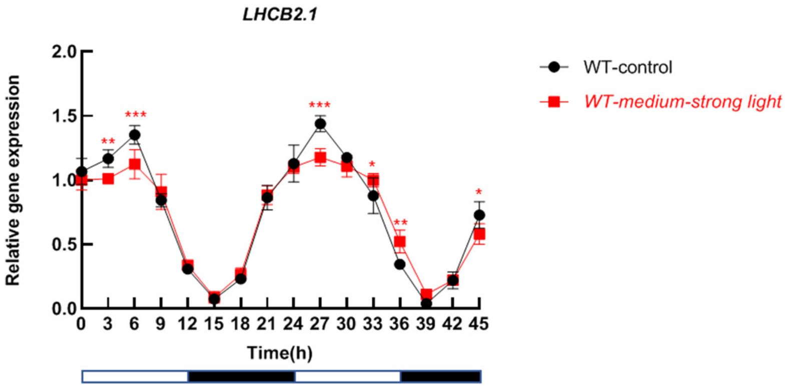

Supplementary Figure S5. Comparative analysis of the rhythmic patterns of *LHCB2.1* under normal light and medium-strong light conditions.

Under normal light and medium-strong light conditions, leaf samples of 2-week-old WT seedlings were taken at different times every 3 hours as indicated. A total of 16 samples were taken. Total RNA was extracted, reverse

transcribed into cDNA, and the expression of annotated genes was estimated by qPCR. *Actin* was used as internal control for normalization. Relative expression is given as the mean value (±SD) of three independent experiments. White bars: light; black bars: dark. The red stars represent that student's t-test of *gun4* versus WT. *: p<0.05, **: p<0.01, ***: p<0.001.

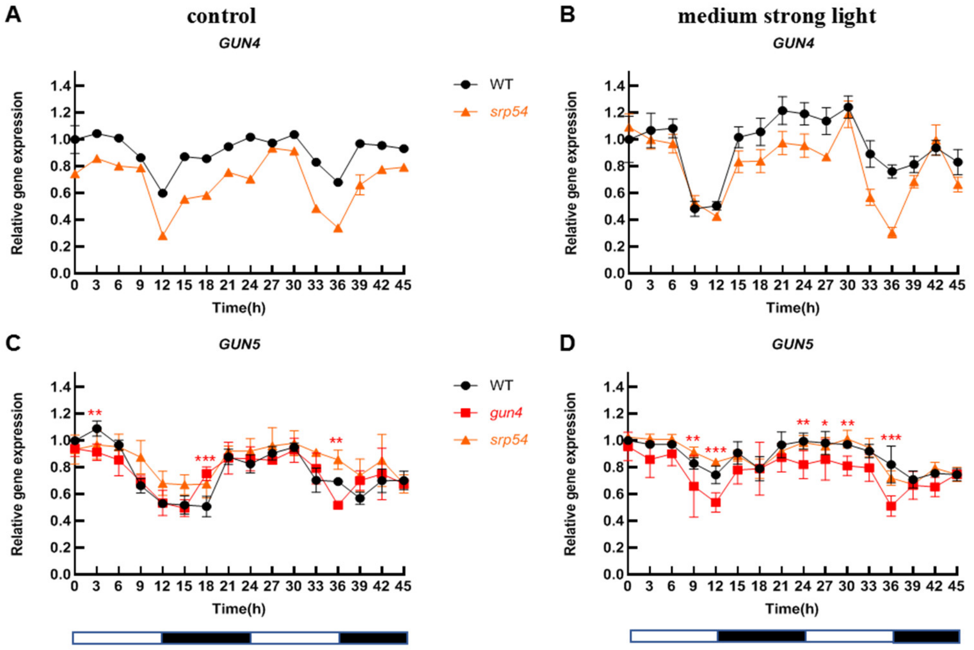

Supplementary Figure S6. Analysis of the rhythmic patterns of *GUN4* and *GUN5*.

Under normal light and medium-strong light conditions, leaf samples of 2-week-old WT, *gun4*, and *srp54* mutant seedlings were taken at different times every 3 hours as indicated. A total of 16 samples were taken. Total RNA was extracted, reverse transcribed into cDNA, and the expression of annotated genes was estimated by qPCR. *Actin* was used as internal control for normalization. Relative expression is given as the mean value (±SD) of three independent experiments. White bars: light; black bars: dark. The red stars represent that student's t-test of *gun4* versus WT. *: p<0.05, **: p<0.01, ***: p<0.001.

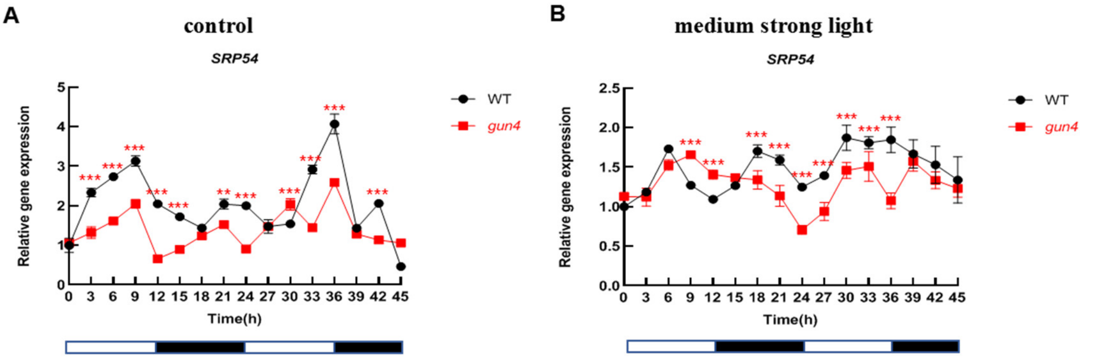

Supplementary Figure S7. Analysis of the rhythm patterns of *SRP54*.

Under normal light and medium-strong light conditions, leaf samples of 2-week-old WT, *gun4*, and *srp54* mutant seedlings were taken at different times every 3 hours as indicated. A total of 16 samples were taken. Total RNA was extracted, reverse transcribed into cDNA, and the expression of annotated genes was estimated by qPCR. *Actin* was used as internal control for normalization. Relative expression is given as the mean value (±SD) of three independent experiments. White bars: light; black bars: dark. The red stars represent that student's t-test of *gun4* versus WT. *: $p<0.05$, **: $p<0.01$, ***: $p<0.001$.

Supplementary Table S2. List of mutant lines used in this study.

Gene name	Accession number	Mutant line
GUN4	AT3G59400	SALK_011461
SRP54	AT5G03940	SALK_079668
CHAOS	AT2G47450	SALK_047001

Supplementary Table S3. List of oligonucleotides for yeast-two-hybrid analysis.

Gene	Name	Sequence (5' - 3')
GUN4	GUN4BDs	atggccatggaggccgaattcATGGCGACCACAAACTCTCTCC
	GUN4BDa	ccgctgcaggtcgacggatccGAAGCTGTAATTTGTTTTAAACACTCTTT
SRP54	SRP54BDs	atggccatggaggccgaattcATGGAGGCTCTTCAATTTTCCA
	SRP54BDa	ccgctgcaggtcgacggatccGTTACCAGAGCCGAAGCCACG
SRP43	SRP43BDs	atggccatggaggccgaattcATGCAAAAGGTCTTCTTGGCC
	SRP43BDs	ccgctgcaggtcgacggatccTTCATTCATTGGTTGTTGTTGTTG
SRP54	SRP54ADs	gccatggaggccagtgaattcATGGAGGCTCTTCAATTTTCCA

	SRP54ADa	cagctcgagctcgatggatccGTTACCAGAGCCGAAGCCACG
	T7 seq	TAATACGACTCACTATAGGGCG
	AD seq	AGATGGTGCACGATGCACAG
	BD seq	TTTTCGTTTTAAAACCTAAGAGT

Supplementary Table S4. List of oligonucleotides for BiLC analysis.

Gene	Name	Sequence (5' - 3')
GUN4	GUN4nLUCs	acgggggacgagctcggtaccATGGCGACCACAAACTCTCTC C
	GUN4nLUCa	cgcgtacgagatctggtcgacGAAGCTGTAATTTGTTTTAAAC ACTCTTT
SRP43	SRP43cLUCs	tacgcgtcccggggcggtaccATGCAAAAGGTCTTCTTGGCC
	SRP43cLUCs	acgaaagctctgcaggtcgacTTCATTCATTGGTTGTTGTTGT TG
SRP54	SRP54cLUCs	tacgcgtcccggggcggtaccATGGAGGCTCTTCAATTTTCCA
	SRP54cLUCa	acgaaagctctgcaggtcgacGTTACCAGAGCCGAAGCCACG

Supplementary Table S5. List of oligonucleotides for qPCR analysis.

Gene	Name	Sequence (5' - 3')
CCA1	rtCCA1s	ACCAGTTGATGATCAAGAGG
	rtCCA1a	GGATTGTTGTTGAGGATTCTAC
LHY	rtLHYs	GCCAGGATTCTTGTGCT
	rtLHYa	CTTTGATTGTTTATGTTCCCAAC
TOC1	rtTOC1s	ACTTGACAGAAGAGAGGAAG
	rtTOC1a	TTAAATCAACATTCACGCCG
PRR7	rtPRR7s	CTTGAAGGTAACAACTGTGCTC
	rtPRR7a	CGTTCTAATCAACCCTTTAGCG
PPR9	rtPPR9s	TACCGGTAGAATCAAATCGC
	rtPPR9a	TTTCCTCTTCAACCGGAAC
ELF3	rtELF3s	TCAACAAAGAGTATTTGCTGTTC
	rtELF3a	AGAGGAGGCTTTACCAGA
ELF4	rtELF4s	GAATCTTGACCGGAATTTC
	rtELF4a	CTAGTATTGAGATCAGAATACATG
CO	rtCOs	GTCTAGCAAGTGGGTTTGC
	rtCOa	TACTTGTGCATGAGCTGTG
GLK1	rtGLK1s	GATTTAGAGCACCGCCA
	rtGLK1a	CAGCCGGCGGATTTAAT
GLK2	rtGLK2s	GGCATCATCGAATCATTCAAG
	rtGLK2a	TCCTTGACGTTGTAACTCC
LHCB2.1	rtLHCB2.1s	CTCCGCAAGGTTGGTGTATC
	rtLHCB2.1a	CGGTTAGGTAGGACGGTGTAT

GUN4	rtGUN4s	TGATGGTAGATTCGGATACAGC
	rtGUN4a	CAAGAAGCTTCATCCACTCAAC
GUN5	rtGUN5s	ATCTGAGACAGTGAGGCT
	rtGUN5a	CCTCCTCGTAGACCCAAT
SRP54	rtSRP54s	GATAATGAGGATAGCGAGAGGG
	rtSRP54a	CATCTGTTTTAGCATCTGAGGC
ACTIN	ACTINs	AACTGGGATGATATGGAGAA
	ACTINa	CCTCCAATCCAGACACTGTA
